# A Systematic Review of the Current Status and Quality of Radiomics for Glioma Differential Diagnosis

**DOI:** 10.3390/cancers14112731

**Published:** 2022-05-31

**Authors:** Valentina Brancato, Marco Cerrone, Marialuisa Lavitrano, Marco Salvatore, Carlo Cavaliere

**Affiliations:** 1 IRCCS Synlab SDN, 80143 Naples, Italy; valentina.brancato@synlab.it (V.B.); direzionescientifica.irccssdn@synlab.it (M.S.); carlo.cavaliere@synlab.it (C.C.); 2 School of Medicine and Surgery, University of Milano-Bicocca, Via Cadore 48, 20900 Monza, Italy; marialuisa.lavitrano@unimib.it

**Keywords:** glioma, differential diagnosis, radiomics, radiomics quality score, texture analysis

## Abstract

**Simple Summary:**

Gliomas can be difficult to discern clinically and radiologically from other brain lesions (either neoplastic or non-neoplastic) since their clinical manifestations as well as preoperative imaging features often overlap and appear misleading. Radiomics could be extremely helpful for non-invasive glioma differential diagnosis (DDx). However, implementation in clinical practice is still distant and concerns have been raised regarding the methodological quality of radiomic studies. In this context, we aimed to summarize the current status and quality of radiomic studies concerning glioma DDx in a systematic review. In total, 42 studies were selected and examined in our work. Our study revealed that, despite promising and encouraging results, current studies on radiomics for glioma DDx still lack the quality required to allow its introduction into clinical practice. This work could provide new insights and help to reach a consensus on the use of the radiomic approach for glioma DDx.

**Abstract:**

Radiomics is a promising tool that may increase the value of imaging in differential diagnosis (DDx) of glioma. However, implementation in clinical practice is still distant and concerns have been raised regarding the methodological quality of radiomic studies. Therefore, we aimed to systematically review the current status of radiomic studies concerning glioma DDx, also using the radiomics quality score (RQS) to assess the quality of the methodology used in each study. A systematic literature search was performed to identify original articles focused on the use of radiomics for glioma DDx from 2015. Methodological quality was assessed using the RQS tool. Spearman’s correlation (ρ) analysis was performed to explore whether RQS was correlated with journal metrics and the characteristics of the studies. Finally, 42 articles were selected for the systematic qualitative analysis. Selected articles were grouped and summarized in terms of those on DDx between glioma and primary central nervous system lymphoma, those aiming at differentiating glioma from brain metastases, and those based on DDx of glioma and other brain diseases. Median RQS was 8.71 out 36, with a mean RQS of all studies of 24.21%. Our study revealed that, despite promising and encouraging results, current studies on radiomics for glioma DDx still lack the quality required to allow its introduction into clinical practice. This work could provide new insights and help to reach a consensus on the use of the radiomic approach for glioma DDx.

## 1. Introduction

Gliomas are the most common primary brain tumor, which originate in the glial cells, including astrocytes, oligodendrocytes, and ependymal cells [1]. According to the World Health Organization (WHO) grading system, gliomas are categorized into grades 1 to 4. Except for pilocytic astrocytoma (WHO grade 1), all the WHO 2–4 gliomas are malignant tumors [2].

Although comprising less than 2% of all newly diagnosed cancers, gliomas are associated with substantial mortality and morbidity. Of these, glioblastoma multiforme (GBM) is the most aggressive and lethal glioma and accounts for 70–75% of all gliomas [3].

Concerning clinical aspects, glioma predominantly manifests with neurological signs, which can also be encountered in other neoplastic and nonneoplastic lesions such as brain inflammation, abscess, lymphoma, or brain metastasis [4,5].

Brain imaging has a fundamental role in glioma management, for establishing an accurate diagnosis, classification, surgical planning, and post-treatment follow-up. Commonly, a brain computed tomography (CT) scan is the initial imaging modality used to diagnose glioma, which presents as a hypodense lesion, possibly showing rim enhancement following contrast agent injection. Despite providing important anatomical information, CT is usually followed by magnetic resonance imaging (MRI), which is generally considered superior to CT in terms of contrast resolution and can provide complementary information [6,7]. MRI with gadolinium contrast enhancement is considered the gold standard imaging method for assessing brain tumors. It provides information on location, mass effect, peritumoral edema, and contrast-enhancement [7]. However, advances in imaging techniques have allowed for a more detailed characterization of tumor characteristics and for a deeper investigation of glioma pathophysiological aspects. Advanced MRI sequences such as perfusion, advanced diffusion protocols [8], and susceptibility weighted imaging, as well as positron emission tomography (PET) scans with specific radiotracer, have emerged as valuable tools to inform clinical decision making and provide a non-invasive way to help in glioma management [9]. 

Nevertheless, beyond what concerns the overlapping clinical manifestations, gliomas can be difficult to discern radiologically from other brain lesions (either neoplastic or non-neoplastic) since their preoperative imaging features often overlap and appear misleading. Because certain lesions require nonoperative treatments, it is necessary to distinguish them from gliomas, and this constitutes a serious clinical challenge affecting both surgical planning and follow-up treatment.

For example, primary central nervous system lymphoma (PCNSL) is a common brain lesion that has shown an increase in occurrence in recent decades as the number of immunosuppressed and immunocompetent patients has increased. On MRI, PCNSL and high-grade gliomas share structural overlaps and anatomical similarities, both of which show contrast-enhancing lesions with peritumoral edema [10]. Similarly, distinguishing a glioma from brain metastasis is another clinical challenge, not only because of the similar symptoms of these conditions but also due to their very similar appearance on conventional MRI sequences as solitary, highly enhancing brain tumors surrounded by a T2-hyperintense edema [4,11].

Furthermore, despite the great spectrum of imaging available, a wide range of brain non-neoplastic disorders can mimic a brain tumor, both clinically and radiologically, posing a potential pitfall for physicians involved in patient care. For example, distinguishing brain parenchyma inflammation from grade II glioma can be difficult for neuroradiologists since both inflammation and glioma appear on conventional MRI sequences as lesions with a mass effect. Moreover, they have similar properties on specific sequences, such as hypointensity on T1-w, hyperintensity on T2-w, and no enhancement on postcontrast T1-CE [4,12].

As a result, there is a continued need for more accurate pre-operative glioma differential diagnosis (DDx), which may be conducted non-invasively with more advanced imaging techniques or through artificial intelligence methods [13,14].

In light of the above, the use of radiomics could be extremely helpful for non-invasive glioma DDx since it uses a voxel-by-voxel approach to convert the sparse imaging data into big data (histogram, texture, and transformed features). The concept behind radiomic is that biomedical imaging derived from medical images (e.g., CT, MRI, and PET) contains hidden information that can be discovered by quantitative image analyses and used to obtain pathophysiological information so as to supplement data held by the radiologist [15,16].

Using advanced mathematical algorithms, radiomics has advantages in exploiting more tumor features that cannot be recognized by the naked eye [17]. The basic principle of radiomics is that a pathological process that alters the tissue modifies the intensity and distribution of the pixels, which will be reflected in different values of textural features with respect to those of the normal tissue and/or tissues affected by other diseases [18].

In neuro-oncology, these features can potentially be used for DDx of newly diagnosed cerebral lesions suggestive of brain tumors [19]. 

In the last decade, radiomics studies aiming at differentiating gliomas from other intracranial diseases have substantially increased, with many demonstrating the power of radiomic features for distinguishing between gliomas and metastases, as well as gliomas and PCNLS, and also non-neoplastic brain diseases [12,20,21]. Nevertheless, the current use of radiomics in glioma differentiation is rather confined to the academic literature, with no research translating to clinical applications, thus generating doubts among clinicians about the validity of radiomics in this field. This is owing in part to a general lack of efficient and effective strategies for translation of imaging biomarkers into clinical practice. In response to the great need for a qualified reporting, standardized evaluation of the performance, reproducibility, and clinical utility of radiomics, a system of metrics to determine the validity and completeness of radiomics studies was developed by Lambin et al. in the form of the radiomics quality score (RQS) [15]. The RQS is a modality-independent tool developed to assess the methodological quality of studies using radiomics. It is based on 16 items that reward and penalize the methodology and analyses of a radiomics study, thus encouraging best scientific practice.

Given the above, the aim of our study was to summarize the current status of radiomic studies concerning glioma DDx, evaluating the radiomics analysis conducted in previous publications by means of the RQS. Our intention was to promote the quality of radiomics research studies in glioma DDx, analyzing its feasibility for medical decision making, and triggering integrated clinical and advanced imaging analyses.

## 2. Materials and Methods

### 2.1. Search Strategy and Selection Criteria

A systematic search for all published studies using radiomics for glioma DDx was conducted. Three of the most relevant scientific electronic databases (PubMed, Web of Science, Google Scholar) were comprehensively explored and used to build the search. Only studies published since 2015 were selected. The last search was performed on 1 March 2022. The search strategy included the key terms listed in Supplementary Materials. The literature search was restricted to English-language publications and studies of human subjects. 

Two reviewers, after having independently screened identified titles and abstracts, assessed the full text of articles that evaluated the use of a radiomics approach for glioma DDx with respect to other diseases and were not review articles. For articles meeting these criteria with full text available, the following further selection criteria had to be fulfilled: involvement of patients in confirmed diseases by pathology and/or surgery and/or overall analysis combined with medical history, clinical symptoms, and various imaging data; presence of information about imaging protocol. Studies were excluded if they aimed at differentiating between different types of glioma (this kind of classification cannot be considered as “DDx” since it falls within the “grading” task).

### 2.2. Planning and Conducting the Review

After the selection procedure, selected articles were analyzed by two reviewers, and data useful for conducting the systematic review were collected in a predesigned sheet. Extracted data will include the following: study characteristics (first author name, publication year, scientometric indexes, namely, Impact Factor (IF), 5-years IF, CiteScore, H-index, first author IF with and without self-citations, study design, in particular prospective or retrospective, number of included patients), diseases involved in the DDx task, imaging modalities used for radiomic feature extraction, information on the ROI placement, software for radiomic feature extraction, number and feature type, feature selection methods (if used), classification methods, validation methods (if used), information on whether models were applied to a separate test or validation datasets, highest accuracy/most important results, and main findings.

Studies were classified and analyzed according to the purpose they had, and in particular to diseases evaluated other than glioma in the DDx task. This systematic review was conducted in accordance with the Preferred Reporting Items for Systematic Reviews and Meta-Analyses (PRISMA) statement (see Supplementary Materials for PRISMA Checklist) [22]. This systematic review has been registered on the Centre for Open Science’s Open Science Framework (OSF) (osf.io/3ksa9).

### 2.3. Quality Assessment Using RQS Evaluation

The methodological quality of each study was evaluated by two reviewers independently using the Radiomic Quality Score (RQS) [15]. Any disagreement was resolved by consensus. RQS tool is composed of 16 items structured to assess various crucial steps in the workflow of radiomics analyses. In particular, a maximum of 36 points can be assigned to each study: up to 2 points for the first (a single item, namely “Image protocol quality”), up to 3 points for the second (3 items, specifically on multiple segmentation strategies, the use of phantoms, and multiple imaging time points), and up to 31 points for the third (12 items, encompassing feature extraction, exploratory analysis design as well as model building and validation) RQS checkpoint (refer to Supplementary Table S1 for RQS checkpoints, items, and points for each item). The total score ranges between −8 and 36 and can be translated into a final 0–100 RQS percentage. Two readers assessed each included study using the RQS and any disagreement was resolved by consensus.

### 2.4. Statistical Analysis

Spearman’s correlation (ρ) analysis was performed to explore whether there was a correlation between RQS and journal metrics (Impact Factor (IF) of the journal at the year of publication, 5-Year IF, CiteScore, and H-index at the year of publication). Moreover, Spearman’s correlation was used to explore the correlation between RQS and H-index of the first author and the year of publication of the study (both with and without self-citations), as well as the association with the number of patients involved in the study and the number of radiomic features investigated. Finally, to explore whether there was a difference in RQS according to the clinical purpose of the study, a subgroup analysis was performed using Kruskal–Wallis. In case of significance, Wilcoxon rank-sum post hoc tests with Bonferroni correction were carried out on each pair of groups. The significance level was set at 0.05. All statistical analysis was performed using SPSS (version 27) (SPSS Inc., Chicago, IL, USA).

## 3. Results

### 3.1. Study Selection

**Figure 1:**
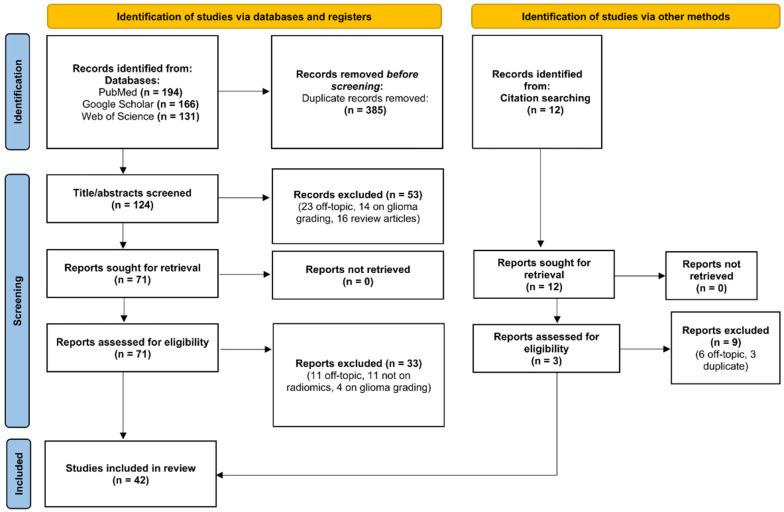
Preferred Reporting Items for Systematic Reviews and Meta-Analyses (PRISMA) flow diagram of included studies.

A total of 491 articles were retrieved by searching scientific electronic databases. After removal of duplicates, there were 124 articles left for investigation. By scanning the title and abstract of these records, 53 records were excluded because they clearly did not match the inclusion criteria (23 were off-topic, 14 were on glioma grading, 16 were review articles). A total of 71 articles were evaluated on their full text. Of these articles, 19 records were excluded based on the inclusion criteria (15 were off-topic, 11 were not on radiomics, 4 were on glioma grading). An additional 12 articles were found through references of selected articles or pre-existing review/systematic review/meta-analyses, of which 3 were included in our study. Finally, 41 records were included for qualitative synthesis. The PRISMA flow diagram of included studies according to the inclusion and exclusion criteria is presented in Figure 1.

### 3.2. Characteristics of Included Studies

Characteristics of the 42 selected articles selected are reported in Table 1. The median number of patients (±absolute deviation) was 107.5 ± 76.64. Study designs were 4.8% (2/42) prospective and 95.2% (39/42) retrospective. All studies except one investigated the power of radiomic features arising from MRI for DDx. Only two investigated radiomic features from 18FDG-PET [23,24] and only one investigated the power of CT radiomics for glioma DDx [25]. A total of 20 studies focused on radiomics for DDx of primary nervous system lymphoma (PCNSL) and glioma (47.6%), with all but one involving IV glioma grade (GBM) patients. In total, 16 studies explored the diagnostic feasibility of radiomic features for DDx of glioma and metastases (38.1%), with all but three studies involving IV glioma grade (GBM) patients. One study investigated the power of radiomic features for DDx of GBM, PCNSL, and metastasis and was discussed separately (GBM vs. PCNLS and GBM vs. MET) [26]. The remaining five studies focused on DDx of glioma and other brain diseases (11.9%). Based on these findings, the following section was divided into three subparagraphs, according to the other diseases involved in the included studies other than glioma.

**Table 1:** Characteristics of included studies. Abbreviations: ST = Study Type; R = Retrospective; P = Prospective; NP = Number of Patients; Seg = Segmentation; FS = Feature Selection; CM = Classification Method; VM = Validation Method. See Supplementary Materials -Section S5- for additional abbreviations.

Authors, Year	ST	Diseases	NP (Type)	Modalities Used for Feature Extraction	Seg	Region for Feature Extraction	Software Used for Feature Extraction	Features Number (Type)	FS	CM	VM	Model Applied to a Separate Dataset?	Most Important Result	Main Findings
Choi et al., 2016 [27]	R	PCNSL, GBM	42 (19 GBM, 23 PCNSL)	CE-T1WI (IAUC), ADC	S, 3D	CE tumor (no necrosis)	MIPAV	3 (histogram)	no	multivariate model	LOOCV	no	AUC = 0.886	The IAUC may be a useful parameter together with ADC for differentiating between PCNSL and atypical GBM.
Alcaide-Leon et al., 2017 [28]	R	PCNSL, glioma	106 (35 PCNSL, 71 glioma)	CE-T1WI	M, 3D	CE tumor	NR	153 (first-order, second-order texture metrics)	SVM— F-statistic	SVM	nested 10-fold CV	no	AUC = 0.87	SVM based on textural features of CE-T1WI is not inferior to expert human evaluation in PCNSL/glioma differentiation.
Chen et al., 2017 [29]	P	PCNSL, GBM	96 (30 PCNSL, 66 GBM)	CE-T1WI	A, 3D	whole tumor	NR	16,384 (SIFT features)	*t*-test, GA	SVM	LOOCV	yes	AUC = 0.991	SIFT method produced more competitive PCNSL and GBM differentiation performance by using conventional MRI.
Wu et al., 2017 [30]	R	PCNSL, GBM	102 (32 PCNSLs, 70 GBMs)	T2WI, CE-T1WI	A + S for small tumors, 3D	CE tumor and peritumoral edema	Matlab	NR (Deep learning features)	sparse representation-based feature selection method	sparse representation classification	LOOCV	yes	Acc = 98.51%	The SRR system had superior PCNSL/GBM differentiation performance compared to advanced imaging techniques.
Artzi et al., 2019 [31]	R	GBM, MET	439 (212 GBM, 227 MET)	CE-T1WI	S, 3D	CE tumor	Matlab R2017a	757 (Location, first-order, second-order, morphological, wavelet)	NCA, PCA	SVM, kNN, DT, ensemble classifiers, BoF	5-fold CV	yes	AUC = 0.85	GBM/MET differentiation showed a high success rate based on postcontrast T1W. GBM/MET subtypes classification may require additional MRI sequences.
Kang et al., 2018 [32]	R	PCNSL, GBM	196 (119 GBM, 77 PCNSL)	CE-T1WI, ADC	S, 3D	CE tumor	Matlab R2014b	1618 (first-order, shape, texture, wavelet)	12 feature selection methods	KNN, NB, DT, LDA, RF, AB, boosting, linear SVM, radial basis function SVM	10-fold CV	yes	AUC = 0.983	The diffusion radiomics model yielded a better diagnostic performance than conventional radiomics or single advanced MRI in identifying atypical PCNSL mimicking GBM.
Kim et al., 2018 [21]	R	PCNSL, GBM	143 () 86 (78 GBM, 65 PCNSL)	T2w, FLAIR, CE-T1WI, DWI	S, 3D	CE tumor and whole (enhancing or non-enhancing) tumor plus peritumoral edema	Matlab	127 (16 shapebased, 57 histogram-based, and 54 texture-based)	mRMR, LASSO	3 classifiers: logistic classifier, SVM, RF	10-fold CV	yes	AUC = 0.979 in the discovery cohort and 0.956 in the validation cohort	Radiomics features derived from multi-parametric MRI can be used to differentiate PCNSL from glioblastoma effectively.
Kunimatsu et al., 2018 [33]	R	PCNSL, GBM	60 (16 PCNSL, 44 GBM)	CE-T1WI	S, 2D	CE tumor	R	67 (first-order, second-order features)	ICC, *t*-test	PCA	no	no	NR	Among MRI-based textures, first-order entropy, median, GLRLM-based run length non-uniformity, and run percentage are considered to enhance differences between GBM and PCNSL.
Nakagawa et al., 2018 [34]	R	PCNSL, GBM	70 (45 GBM, 25 PCNSL)	T2, rCBV, CE-T1WIs, ADC	M, 2D	whole tumor	LIFEx	48 (12 for each sequence) (histograms and texture parameters)	not performed	LR, multivariate XGBoost	10-fold CV	no	AUC = 0.98	mpMRI radiomics model outperformed conventional cut-off method and the board certified radiologists in distinguishing GBM from PCNSL.
Suh et al., 2018 [35]	R	PCNSL, GBM	77 (54 PCNSL, 23 non-necrotic atypical GBM)	post-contrast T1- and T2-weighted, and FLAIR; ADC (10th percentile)	S, 3D	CE tumor, NE tumour tissue and edema	PyRadiomics	6366 (shape, volume, first-order, texture, and wavelet)	*t*-test, recursive feature elimination	RF	nested CV	no	AUC = 0.921	The radiomics model yields a better diagnostic performance than human radiologists and ADC values.
Xiao et al., 2018 [36]	R	PCNSL, GBM	82 (22 PCNSL, 60 GBM)	T1WI, CE-T1WI	M, 3D	CE tumor and intratumoral cysts	PyRadiomics	105 (92 texture features and 13 geometric features)	Weka CfsSubsetEval	ROC analysis; NB, SVM, LR, RF	10-fold CV	no	AUC = 0.90 for NB; Acc = 0.92 for SVM	MRI-based 3D texture analysis has potential utility for preoperative GBM/PCNSL discrimination.
Bao et al., 2018 [37]	R	GBM, PCNSL	20 (9 PCNSL, 11 GBM)	rCBV, ADC	S, 3D	CE tumor (no cysts and necrosis)	nordicICE	11 (histogram)	no	Multivariate LR	no	no	AUC = 0.97	Whole-tumor histogram analysis of nCBV and ADC was able to differentiate between GBM and PCNSL.
Chen et al., 2019 [38]	R	GBM, MET	134 (77 gbm, 58 MET)	CE-T1WI	M, 3D	CE tumor	LIFEx	43 (shape, first-order, texture)	five selection methods: RF, LASSO, XGBoost, GBDT	LDA, SVM, RF, KNN, Gaussian NB, LR.	4-fold	yes	AUC = 0.80	Radiomic-based machine learning has potential to be utilized in differentiating GBM from MET.
Dong et al., 2019 [39]	R	GBM, MET	120 (60 GBM, 60 MET)	T1W, T2W, CE-T1WI	M, 3D	peri- enhancing oedema	PyRadiomics	321 (shape, first-order, texture)	ICC, Boruta algorithm	DT, SVM, NN, NB, KNN	10-fold CV	yes	AUC from 0.70 to 0.76, for the training dataset, and from 0.56 to 0.64 for the validation data set	Combined use of classifiers could confer extra benefits for GBM/MET differentiation.
Kong et al., 2019 [23]	R	PCNSL, GBM	77 (24 lymphoma, 53 GBM)	SUV map, SUVncc map, SUVnbm map	M, 3D	whole tumor	PyRadiomics	107 (shape, first-order, texture)	ICC	ROC analysis	10-fold CV	no	AUC = 0.998	18F-FDG-PET-based radiomics is a reliable noninvasive method to distinguish PCNSL from GBM.
Kumimatsu et al., 2019 [40]	R	PCNSL, GBM	76 (55 GBM, 21 PCNSL)	CE-T1WI	S, 2D	CE tumor	R	67 (texture)	ICC, PCA	KNN, DT, LDA, SVM	6-fold CV	yes	AUC = 0.99 on training set; Acc = 75% on test data	Radiomics MRI may provide complementary diagnostic information on routine brain MRI.
Petrujkic et al., 2019 [41]	R	GBM, MET	55 (30 GBMs and 25 solitary MET)	T2W, SWI, CE-T1WI	M, 3D	CE tumor	ImageJ	14 (Euclidian, fractal, texture (GLCM))	no	ROC analysis	no	no	AUC = 0.908	Texture features are more significant than fractal-based features in GBM/MET differentiation.
Qian et al., 2019 [42]	R	GBM, MET	412 (242 GBM, 170 solitary brain MET)	T1W, T2W, CE-T1WI	M, 3D	CE tumor	PyRadiomics	1303 (shape, first-order, texture, square, square root, logarithm, exponential, LoG, wavelet)	12 methods (filter, wrapper, embedded methods).	7 supervised machine-learning algorithms	5-fold CV	yes	AUC ≥ 0.95 in the training set; AUC = 0.90 in the test set	Radiomic machine-learning technology could help in differentiating GBM from MET preoperatively.
Wang et al., 2019 [43]	R	PCNSL, GBM	109 (28 PCNSL, 81 GBM)	T2W	M, 2D	CE tumor (no hemorrhage, necrosis, cysts, non- enhancement)	ImageJ	5 (texture)	no	binary logistic regression	no	no	AUC = 0.917	The texture features of T2WI and conventional imaging findings may be used to distinguish GBM from PCNSL.
Yun et al., 2019 [44]	R	PCNSL, GBM	195 (119 GBM, 76 PCNSL)	CE-T1WI	S, 3D	CE tumor	Matlab	936 (first- order, texture, wavelet)	Metric 1: mRMR, CFS, backward elimination; Metric 2: MLP network	Metric 1: SVM, the boosted generalized linear mixed model, regularized RF; Metric 2: MLP network	Metric 1: 10 fold CV Metric 2: 10 fold CV	yes	AUC > 0.82	A combination of radiomic features and MLP network classifier serves a high-performing and generalizable model for PCNSL/GBM DDx.
Bae et al., 2020 [45]	R	GBM, MET	248 (159 GBM, 89 MET)	CE mask on CE-T1WI, CE mask on T2WI, and PT mask on T2WI	S, 3D	CE tumors, non- enhancing T2 hyperintense tumors	PyRadiomics	265 (first- order, texture)	five methods for feature selection	KNN, NB, RF, AdaBoost, L-SVM, SVM using radial basis function kernel, LDA; Multi input DNN	10-fold CV	yes	AUC = 0.95	The results demonstrated that deep learning using radiomic features can be useful for distinguishing GBM/MET.
Dastmalchian et al., 2020 [46]	P	GLIOMAS, MET	31 (17 GBM, 6 LGG, 8 MET)	T1 and T2 maps	M, 2D	CE tumors and peritumoral white matter	Matlab	39 (texture (GLCM, GLRLM))	Spearman correlation filter, Wilcoxon	ROC analysis	no	no	AUC = 0.952 (LGG vs. MET); AUC = 0.877 (GBM vs. MET)	Texture analysis of MRF-derived maps can improve our ability to differentiate glioma from GBM.
Chen et al., 2020 [47]	R	GBM, PCNSL	138 (76 GBM, 62 PCNSL)	CE-T1WI	A, 3D	whole tumor	lifeX	43 (histogram, shape, texture)	distance correlation, RF, LASSO, XGBoost, GBDT	LDA, SVM, LR	validation set, 100 train-validation repetition times	yes	AUC = 0.98	Radiomics-based machine-learning algorithms potentially have promising performances in differentiating GBM from PCNSL.
Dong et al., 2020 [48]	R	EP, MB	51 (24 EPs, 27 MB)	CE-T1W, ADC	S, 3D	CE tumors	3D Slicer	188 (shape, first-order, texture)	*t*-test, multivariable LR, univariate analysis screening	kNN, AdaBoost, RF, SVM	10-fold CV	no	AUC = 0.91	The combination of radiomics and machine-learning approach on 3D multimodal MRI could well distinguish EP and MB.
Oritz-Ramon et al., 2020 [20]	R	GBM, MET	100 (50 MET, 50 GBM)	T1w	M, 2D	CE tumors	Matlab	88 (histogram, texture, and local binary patterns)	ICC, MWW, MIC, Relief-F	RF, SVM, KNN, NB, MLP	nested CV	no	AUC = 0.896	The proposed radiomics MRI approach is able to discriminate between GBM and BM.
Xia et al., 2020 [49]	R	PCNSL, GBM	240 (129 GBM, 111 PCNSL)	FLAIR, DWI, CE-T1WI, ADC	M, 3D	Tumor tissue and peritumoral edema	PyRadiomics	851 (shape, first-order, texture, wavelet)	ICC, Spearman correlation filter, Mrmr, LASSO	LASSO	10-fold CV	yes	AUC = 0.943	The model combining MP-MRI and radiologists’ diagnoses had superior performance to the radiologists alone.
Zhou et al., 2020 [50]	R	MB, EP, PA	288 (111 MB, 70 EP, 107 PA)	CE-T1WI, T2WI, DWI, ADC maps	S, 3D	CE and non-CE tumor and peritumoral edema	Matlab	3087 (shape, first-order, texture)	multiclass classification: TPOT; binary classification: 13 different feature-selection methods	multiclass classification: TPOT; binary classification: 10 machine learning classifiers	5-fold CV	yes	AUC = 0.84–0.94	Automatic machine learning based on routine MRI classified pediatric posterior fossa tumors with high accuracy compared with manual expert pipeline optimization and qualitative expert MRI review.
Csutak et al., 2020 [51]	R	GLIOMAS, MET	42 (16 HGGs, 26 MET)	T2WI	S, 3D	peritumoral region	MaZda	NS	Fisher, POE + ACC	univariate analysis (*t*-test), ROC analysis, multiple regression	no	no	75–87.5% sen, 53.85–88.46% spec (univariate); 100% sens, 66.7% spec (multivariate)	Texture analysis can provide a quantitative description of the peritumoral zone encountered in solitary brain tumors.
Xia et al., 2021 [52]	R	GBM, PCNSL	289 (136 PCNSL, 153 GBM)	CE-T1WI, FLAIR, ADC	M, 3D	whole tumor	PyRadiomics	851 (NS)	mRMR, LASSO	Logistic regression	5-fold CV	yes	AUC = 0.865	A CNN model can differentiate PCNSL from GBM without tumor delineation, and comparable to the radiomics models and radiologists
Bathla et al., 2021 [53]	R	GBM, PCNSL	94 (34 PCNSL, 60 GBM)	CE-T1WI, FLAIR, ADC	S, 3D	CE tumor and surrounding edema	PyRadiomics	107 (shape, first-order, texture)	linear combination filter, high correlation filter, PCA	12 classifiers (linear, non-linear, and ensemble)	5-fold repeated CV	no	AUC = 0.98	Radiomics-based diagnostic performance of various machine learning models for differentiating glioblastoma and PCNSL varies considerably.
Priya et al., 2021 [26]	R	GBM, PCNSL, MET	253 (120 MET, 40 PCNSL, 93 GBM	T1W, T2W, FLAIR, ADC map, T1-CE	S, 3D	whole tumor, CE tumor, necrosis, peritumoral edema	PyRadiomics 3.0	107 (shape, first-order, texture)	linear combination filter, a high correlation filter, PCA	12 classifiers (linear, non-linear, and ensemble)	5-fold repeated CV	no	AUC = 0.91 for mpMRI, AUC = 0.90 for T1-CE	T1-CE is the single best sequence with comparable performance to that of MP-MRI.
De Causans et al., 2021 [54]	R	GBM, MET	143 (71 GBM, 72 BM)	post-contrast T1-CE	S, 3D	CE tumor and necrotic region	PyRadiomics 2.1.2	100 (shape, first-order, texture)	9 feature scaling methods	16 classifiers	stratified 5-fold CV	yes	AUC = 0.92 in the training CV set, AUC = 0.85 in the test set	The proposed diagnostic support system helps in differentiating solitary BM from GBM with high diagnosis performance and generalizability.
Zhang et al., 2021 [24]	R	GBM, MET	100 (50 GBM, 50 MET)	CE-T1WI, T2WI, ADC, 18F-FDG PET	S, 3D	CE tumor and perifocal edema	PyRadiomics	4424 (shape, first-order, texture, LoG, wavelet)	*t*-test, PCA	partial least squares	5-fold CV	yes	AUC = 0.98 in TS and 0.93 in VS	An integrated radiomics model incorporating DWI and F-FDG PET improved performances of GBM/MET differentiation.
Han et al., 2021 [55]	R	GBM, MET	350 (182 GBM, 168 MET)	CE-T1WI	M, 3D	CE tumor	PyRadiomics v3.0	841 (shape, first-order, texture)	CMIM, MR R, DISR, Fisher, relief, MCFS, RFS	LR, SVM, DT, RF	5-fold CV	yes	AUC = 0.764	The combination models incorporating the radiomics signature and clinical-radiological characteristics were superior to the clinical-radiological models in differentiating between GBM and MET.
Han et al., 2021 [12]	R	GLIOMA, INFLAMMATION	57 (39 grade II glioma, 18 inflammation)	T1W and T2W	M, 3D	whole tumor	MATLAB 2014b	45 (shape, global, texture)	two-sample *t*-test, LASSO	linear regression	10-fold CV	yes	AUC = 0.98–0.988 in primary cohort and 0.950, 0.925 in validation cohort	The radiomics signature helps to differentiate inflammation from grade II glioma and improved performance compared with experienced radiologists.
Priya et al., 2021 [56]	R	GBM, MET	120 (60 GBM, 60 MET)	T1W, T2W, FLAIR, ADC, CE-T1WI	S, 3D	CE tumor + necrosis, surrounding edema	PyRadiomics	107 (shape, first-order, texture)	linear combinations filter, a high correlation filter, PCA	20 different models grouped into: linear classifiers, non-linear classifiers, and ensemble classifiers	5-fold CV	no	AUC = 0.951	Radiomics based machine learning can classify GBM and IMD with excellent diagnostic performance. The performance of mpMRI and single FLAIR or combined T1-CE and FLAIR sequence is comparable.
Priya et al., 2021 [57]	R	PCNSL, GBM	97 GBM and 46 PCNSL	T1W, T2W, FLAIR, ADC, CE-T1WI	M, 3D	CE tumor + necrosis	TexRAD	72 (histogram first-order (LoG filtered))	full-features, correlation, PCA	12 models grouped into: linear classifiers, non-linear classifiers, and ensemble classifiers	5-fold CV	no	LASSO model with correlation filter as selection method: AUC = 0.914	T1-CE derived first-order texture analysis can differentiate between GBM and PCNSL with good diagnostic performance.
Sartoretti et al., 2021 [58]	R	GLIOMAS, MET	48 (21 gliomas, 27 MET)	APTw	M; 3D	whole tumor	PyRadiomics	110 (first-order features; shape features; texture features)	ICC, correlation-based (CfsSubsetEval by Weka)	Multilayer perceptron classifier, Random Forest	10-fold CV	yes	AUC = 0.797	The use of radiomics for APTw imaging is feasible and the differentiation of primary glial brain tumors from metastases is achievable with a high degree of accuracy.
Su et al., 2021 [59]	R	GBM, MET	225 (157 GBM, 98 solitary brain MET)	CE-T1WI	M; 3D	CE tumor	AK software	396 (first-order features; shape features; texture features)	ICC, Mmrmr, LASSO	logistic regression	10-fold CV	yes	AUC of 0.82 and 0.81 in the training and validation cohort to distinguish between GBM and solitary brain MET	The radiomics model might be a useful supporting tool for the preoperative differentiation of GBM from solitary brain MET, which could aid pretreatment decision making.
Xiao et al., 2021 [60]	R	GBM, BRAIN ABSCESS	118 (86 nGBM, 32 BRAIN AB)	CE-T1WI, T2 FLAIR	S, 3D	Peritumoral edema, tumor	PyRadiomics	1004 (shape, first-order, texture, LoG, wavelet)	LASSO, PCA	logistic regression, RF	5-fold CV with 1000 iterations	yes	AUC = 0.97	The radiomic features combined with the peritumoral edema/tumor volume ratio provided the prediction model with the greatest diagnostic performance.
Bo et al., 2021 [61]	R	CYSTIC GLIOMA, BRAIN ABSCESS	188 (102 BRAIN ABSCESS, 86 CYSTIC GLIOMA)	T1WI and T2WI	M, 3D	whole tumor	PyRadiomics	1000 DTL + 105 radiomic (first-order features; shape features; texture features)	Spearman’s rank correlation, MI	LR, RFC, KNN, and SVM	nested 5-fold CV	yes	AUC = 0.86 in TS and 0.85 in VS	The combination of HCR and DTL features can lead to impressive performance for distinguishing brain abscess from GBM.
Marginean et al., 2022 [25]	R	HGGs, MET	36 (HGGs, n = 17; MET, n = 19)	CT	S, 3D	Peritumoral zone	maZda	275 (GLRLM, wavelet GLCM, histogram, absolute gradient, auto- regressive model)	POE + ACC and Fisher coefficients, Mann– Whitney	Univariate and multivariate regression analysis	no	no	AUC = 0.992	The CT-based TA can be a useful tool for differentiating between HGG and MET.

### 3.3. Radiomics for DDx of Glioma and PCNSL

In total, 21 studies focused on radiomics for DDx of PCNSL and glioma, with all but one [28] involving GBM. Among them, all but one extracted radiomic features from MRI sequences, while the remaining one focused on radiomic features extracted from PET [23].

Among MRI radiomic studies, 6 extracted radiomic features from CE-T1w images. Kunimatsu et al. performed two complementary studies [33,40]. In the first [33], they simply performed image feature extraction and selection and limited the analysis to a principal component analysis to find the predominant features in evaluating the differences between GBM and PCNSL. The training and cross-validation was performed in a subsequent study [40] and found an AUC from 0.87 to 0.99 for the training set and of 0.75 for the testing set.

Xiao et al. [36] compared different supervised classifiers based on T1-CE radiomic features and found that naive Bayes classifier had an AUC of 0.90 for preoperative discrimination of GBM and PCNSL. Similar studies were performed by Priya et al. and Chen et al. [47,57], who found similarly high AUC values for different combinations of classifier models and feature selection techniques. Chen et al. [29] proposed a method based on Scale Invariant Feature Transform features and found that an SVM model based on SIFT features yielded an AUC superior to 0.99 for GBM vs. PCNSL classification task. 

Promising results in DDx between PCNSL and high-grade gliomas were also found by Alcaide-Leon et al. [28], who found that SVM classification based on textural features of T1w-CE is not inferior to expert human evaluation in the differentiation of PCNSL and high-grade gliomas, with similar results in terms of AUC. Notably, their study also involved grade III gliomas other than GBM.

Other studies built prediction models based on radiomic features extracted from multiparametric MRI. In particular, Kim et al. [21] found that a logistic regression-based classifier built starting from CE-T1, T2, and ADC features yielded an AUC superior to 0.95 to distinguish between GBM and PCNSL. Similar classification performances were reached by mpMRI-based classifiers built in studies by Xia et al. and Bathla et al. [49,53]. Interestingly, Pryia et al. found that T1-CE had comparable performance to that of mpMRI-based methods. However, these results were obtained from a three-class problem that also included a group of patients with metastasis. Promising results were also found by Suh et al. [35] in an mpMRI-based radiomic study involving features extracted from CE-T1, T2w, and FLAIR. They found that a random forest classifier built using these features outperformed both ADC values and visual analysis by human radiologists. Findings by Nakagawa et al. [34] were in line with those of Kim et al. However, differently from the previous one, features were extracted from T2, rCBV, CE-T1WIs, and ADC.

Xia et al. [52] found that the combination of CE-T1w and ADC radiomic features showed high diagnostic performances (AUC = 0.94). Moreover, the integration of this model with radiologists’ diagnoses outperformed performances of the radiologists alone. Similar results were obtained by Choi et al. [27], who found that the initial area under the curve derived from CE-T1w could be useful in combination with ADC for differentiating between PCNSL and atypical GBM.

Two studies were performed by the same group [21,44] and were also based on radiomic features extracted from CE-T1w and ADC. In the older one, they evaluated different feature selection methods and machine learning models and found that the combination of recursive feature elimination and a random forest classifier revealed an AUC of 0.984 in the internal and AUC 0.94 in the external validation set. In the more recent study, they utilized a lower number of radiomics features (n = 936 with respect to n = 1618 of the previous one) and applied four different classification metrics, of which two based on radiomic features were extracted from CE-T1w and ADC. Metrics 1 and 2 used radiomic features, and feature selection and classification were optimized with SVM, GLM, or random forest (metric 1) or multilayer perceptron (MLP) network. They found that a deep learning-based MLP network classifier with radiomic features showed the highest performance in differentiating PCNSL from GBM. These results were in line with considerations of Wu et al. [30], who also proposed a radiomic approach based on deep learning and considering CE-T1WI and T2w as MRI sequences. In particular, they proposed a sparse representation-based radiomics system for classifying GBM from PCNSL and found that this approach outperformed traditional radiomics methods.

Among MRI-based studies, Wang et al. and Bao et al. [37] were the only two that did not involve radiomic features extracted from CE-T1. Wang et al. [43] focused only on T2w and found that texture features from T2w could be used for differentiating GBM from PCNSL. However, it should be noted that they considered only 5 textural features. Bao et al. found that the combination of whole-tumor-based histogram features from normalized cerebral blood volume (nCBV) and ADC for contrast-enhancing lesions could be useful for GBM/PCNSL differentiation.

Kong et al. [23] explored a 18F-FDG-PET-based radiomics approach to distinguish PCNSL from GBM. They extracted features from a standardized uptake value (SUV) map, an SUV map calibrated with the normal contralateral cortex (ncc) activity (SUV/ncc map), and an SUV map calibrated with the normal brain mean (nbm) activity (SUV/nbm map). They found that the most discriminative power was achieved by SUV first-order and textural features.

### 3.4. Radiomics for DDx of Glioma and Metastases

A total of 16 studies explored the diagnostic feasibility of radiomic features for DDx of glioma and metastases. All but two of them extracted radiomic features from MRI sequences, while one evaluated features from CT [25] and one extracted features from PET [24]. In all but three studies [25,46,58], the glioma group consisted of patients with grade IV glioma (GBM). Six studies extracted radiomic features from contrast-enhanced T1-weighted MRI scans [20,31,38,54,55,59]. Among them, the largest patient sample was investigated by Artzi et al. [31] (439 patients), who aimed at differentiating GBM and MET subtypes using radiomics analysis based on conventional post-contrast T1w. They tested four different types of machine learning algorithms (both supervised and unsupervised), revealing that SVM was the best (AUC = 0.98). They suggest that classification between glioblastoma and brain metastasis subtypes may require additional MRI sequences with other tissue contrasts. Similar study settings and results can be found in studies by Chen et al. [38], Han et al. [55], and De Causans et al. [54], in which diagnostic models were built based on multiple selection methods and classification algorithms for differentiating GBM from MET. Su et al. [59] aimed to differentiate GBM from primary brain metastases, finding that a radiomics model based on logistic regression might be a useful supporting tool for the preoperative differentiation of GBM from solitary brain MET due to an AUC superior to 80%. Ortiz-Ramon et al. proposed a radiomics MRI approach able to discriminate between GBM and MET with AUC > 80%. Unlike the previous three studies, they used radiomic features extracted from 2D ROIs. 

Dong et al. [39], Qian et al. [42], and Bae et al. [45] investigated multiple classifiers for differentiating between solitary brain MET and GBM by extracting radiomic features from T1w, T2w, and T1-CE. Dong et al. [39] found that features derived from the peri-enhancing oedema region had moderate value in differentiating supratentorial single brain MET from GBM. Qian et al. [42] found more promising results, showing that the clinical performance of the classifier based on SVM and LASSO (>95%) was superior to neuroradiologists’ performances. Bae et al. [45] also investigated multiple feature selection methods and classifiers for differentiating between single brain metastases and GBM. Interestingly, they also compared results from traditional machine learning radiomic approaches with a deep neural network approach. The latter performed better than the best-performing traditional machine learning classifiers or human readers and demonstrated good generalizability in the external validation.

Petrujkić et al. [41] aimed to differentiate GBM and solitary brain metastases of different origin by means of quantitative parameters of fractal and GLCM texture features from T2W, SWI, and CET1 images and found that texture features are more significant than fractal-based features for GBM solitary MET. 

A recent study by Priya et al. [56] also cross-compared multiple radiomics-based machine learning models using features extracted from mpMRI (T1W, T2W, T1-CE, ADC, FLAIR) for DDx of intracranial metastatic disease from GBM and found that FLAIR was the best individual sequence (LASSO-full feature set, AUC 0.951), while for combined T1-CE/FLAIR sequence, adaBoost-full feature set was the best performer (AUC 0.951).

Among studies investigating the value of MRI radiomics features in differentiating brain metastases from both high- and low-grade gliomas (unlike the previously discussed 9 studies involving only GBM), Dastmalchian et al. [46] found that texture features from MRI fingerprinting T1 and T2 maps were able to differentiate brain MET from high- and low-grade glial brain tumors. Notably, they did not build any multivariable model but performed ROC analysis on each feature. Similar results were obtained by Csutak et al., who found that texture parameters from T2w were able to distinguish high-grade gliomas from MET. Notably, they investigated texture analysis of the peritumoral zone [51]. Su et al. evaluated the utility of radiomics for Amide Proton Transfer weighted imaging for the same purpose and in a similar patient cohort. Their classification model based on the random forest classifier achieved an AUC superior to 70%.

Among studies involving other modalities than MRI, Zhang et al. [24] found that an integrated radiomics model incorporating DWI and 18F-FDG PET improved the performance of differentiating GBM from solitary brain metastases. Promising performances (AUC = 0.992) were also obtained from models built using CT-based textural features to differentiate patients with high-grade gliomas from those with solitary brain metastases. However, the patient sample was relatively small (36 patients). 

### 3.5. Radiomics for DDx of Glioma and Other Brain Diseases

Five studies focused on DDx of glioma and other brain tumors, of which two involved paediatric populations. In particular, Dong et al. [48] aimed to investigate the effectiveness of radiomics and machine-learning techniques based on mpMRI in distinguishing the glioma subtype ependymoma from medulloblastoma. They explored different combinations of feature selection and machine learning techniques starting from features extracted from postcontrast T1w images and ADC maps, finding that multivariable logistic regression feature selection combined with the random forest classifier yielded an AUC = 91% for the classification of EP from MB. Zhou et al. [50] aimed to assess the power of machine learning radiomic-based models for differentiating paediatric posterior fossa tumors and involved a larger population of 288 patients. Unlike Dong et al. [48], they extracted features from T2w images, and included patients with the glioma subtype pilocytic astrocytoma, except those with EP and MB in their cohort. Their machine-learning automatic approach revealed an AUC = 94% with an accuracy of 85% for differentiation between MB and non-MB (namely glioma group) and was superior to performances of non-automatic pipeline and qualitative expert MRI review. The third study involved adult patients and aimed to assess the value of MR-based radiomic features arising from T1w and T2w in differentiating brain inflammation from grade II glioma [12]. Their findings were promising, with models’ AUCs superior to 92% and their performances superior to those from experienced radiologists. Finally, the remaining two studies investigated the ability of radiomics to differentiate between gliomas (in particular, necrotic glioblastomas [60] and cystic gliomas [61]) and brain abscess.

### 3.6. Quality Assessment with RQS

**Figure 2:**
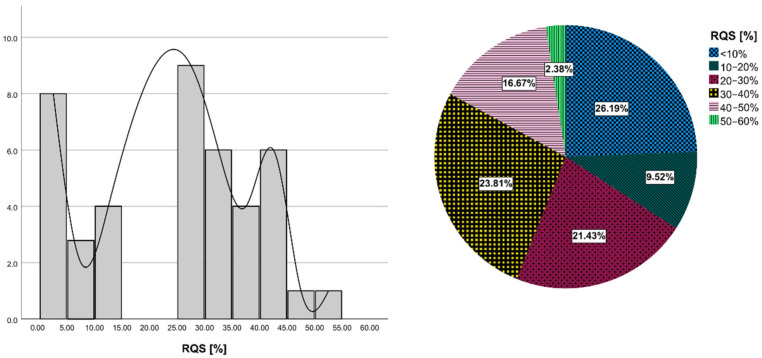
Results of RQS assessment. Histogram plot of row counts of included studies according to RQS percentage (on the **left**). Pie chart of the mean RQS of included studies.

The details of the RQS of all included studies are provided in Supplementary Table S3. The average RQS total score was 8.71 ± 5.67, with the corresponding percentage of 24.21 ± 15.56%, ranging from 0.0 to 52.78% (Figure 2). Concerning the first RQS checkpoint (item 1), all studies provided a comprehensive documentation of imaging protocol, with only two of them scoring the maximum amount of points arising from the usage of a public protocol.

Concerning the second RQS checkpoint (items from 2 to 4), more than half of the studies (57.1%, 24/42) employed multiple segmentation (mainly arising from segmentation by different radiologists), but only five studies satisfied the item of “imaging at multiple time points” and only 3 articles satisfied that of “phantom study”. Regarding items included in the third RQS checkpoint (items from 5 to 16), all but four studies (90.5%) applied feature reduction techniques. Only four studies (9.52%) performed multivariable analysis with non-radiomics features. Only 2 out of 42 included articles (4.76%) were able to detect and discuss biological correlates and only 15 (35.7%) provided a cut-off analysis.

All but one of the studies reported discrimination statistics and their statistical significance, of which all but three applied resampling techniques. Conversely, only 2/42 studies reported calibration statistics, and none of them applied resampling techniques.

In total, 35.7% of the studies (15/42) did not include a validation of their results. Among studies validating their results, only five validated analyses using an external validation cohort and one used two external validation cohorts. Moreover, 8/42 studies compared radiomics models with the specific gold standard and about half of the included studies (21/42) discussed the clinical utility of the developed model by means of decision curve analysis.

Finally, no study included a cost-effectiveness analysis and 11 made code and data publicly available.

### 3.7. Statistical Analysis

There was a significant positive correlation between RQS and journal Impact Factor (ρ = 0.35, *p* = 0.022), number of patients involved (ρ = 0.44, *p* = 0.003), and number of radiomics features (ρ = 0.51, *p* = 0.0009) extracted in the study. On the other hand, weak positive but not significant correlations were found between RQS and 5-year IF, HI of the journal, and of the first author with and without self-citations (ρ = 0.25, ρ = 0.25, ρ = 0.20, and ρ = 0.22, respectively). No statistically significant differences were found between RQS of studies with different aims. Refer to Supplementary Table S2 for details of scientometric indexes of the included studies. 

## 4. Discussion

In this systematic review, we aimed to explore whether radiomics could provide information about the DDx of gliomas, summarizing the current status of the literature research and evaluating the quality of included studies using the RQS tool. The reasons that led us to perform the study are both the urgent need for clinicians to assess alternative noninvasive differential diagnostic tools to ensure an accurate preoperative assessment of intracranial masses (since the lack of a clear diagnosis may therefore lead to invasive procedures that may be inappropriate for the primary disease treatment and could also aggravate a patient’s condition) and the potential power of radiomics for DDx of newly diagnosed cerebral lesions suggestive of brain tumors. 

A total of 42 studies from 2015 onwards were examined. Almost all studies involved machine learning techniques for radiomic analysis, of which two involved unsupervised DNN techniques. Among studies involving supervised machine learning, 24 investigated multiple models combined with multiple feature selection methods and evaluated the combination providing the best result in terms of accuracy.

Despite promising results obtained from each of them (with best AUCs ranging from 0.7 to 0.99), our study revealed that those studies were far from providing definitive conclusions for clinical implementation and widespread use of radiomics for glioma DDx. 

Most of the selected studies explored radiomic approaches for DDx of glioma (mainly GBM) and PCNSL (48%) or GBM and metastases (38%).

Almost all studies investigated radiomic approaches based on MRI. In particular, CE-T1WI sequence was the most investigated since it is the first-line MRI sequence for glioma assessment. Only two studies investigated the ability of PET radiomic features to differentiate gliomas from metastases [24] and glioma from PCNSL [23], and only one study was on CT [25].

The results of RQS have brought out the main positive and negative aspects related to the radiomic workflow followed in each selected study. Mean RQS was 8.71 out 36, with a mean percentage RQS of 24.21%, and this was in line with previously published data regarding prostate, breast, lung, renal, and brain cancer [62,63,64,65]. The lack of a rigorous procedure related to radiomics workflow largely contributed to the low RQS scores of the included studies. 

Concerning RQS checkpoint 1, image protocol was well documented in all studies. Moreover, no studies involved public image protocols which allow reproducibility and replicability. The results of RQS items included in RQS checkpoint 2, more than half of the studies performed multiple segmentations to limit the extent of bias arising from segmentation variability. It is worth noting that the ROI type (2D/3D) and the segmentation method (manual, semi-automatic, automatic) is not uniform across studies. Furthermore, manual or semi-automated image segmentation with manual correction were used in almost all studies, and this limits included studies since it is well known that manual segmentation is time-consuming and both manual and semi-automated segmentation introduce a considerable observation bias and affect studies in terms of intra- and inter-observer variations concerning ROI/VOI delineation [18]. It should also be considered that the area considered for feature extraction was extremely variable across studies. It is worth noting that some studies targeted the enhancing tumor (with or without the inclusion of necrosis and intratumoral cysts) [27,36,54], while others targeted the peritumoral zone [25,51,60].

Notably, no studies determined inter-scanner and inter-vendor variability and collected images at multiple timepoints. On a positive note, considering the third RQS checkpoint, all studies except four performed feature reduction. It is a positive aspect since excessive dimensionality of features negatively affects model performance and could lead to overfitting [66]. 

Another relevant finding emerging from our study was that only two of the included studies were prospectively designed. This constitutes an important limiting factor in radiomic research since a well-designed prospective study can reduce and minimize the potential confounding factors, representing a higher level of evidence for the quality validity (this is the reason why prospective studies are given the highest weighting in the RQS tool (7 points), accounting for around 20% of the full scale).

It is significant that almost half of the reviewed papers did not include a validation of their results, and this negatively affects the risk of false-positive results that prevent the translation of radiomics to clinical practice. On a positive note, among the remaining studies not performing validation with an independent cohort, almost all opted for performing the cross-validation.

Most studies lacked any kind of openness, either in sharing datasets, segmentations, or codes, and this constitutes a significant limitation in terms of verification and reproducibility of the reported findings [67,68].

The same happened for the cost-effectiveness analysis that can evaluate a radiomics prediction model in terms of health economics in case of its application in clinical practice, assuming that a novel predictor should not be more expensive than currently available predictors when accuracy is comparable and comparing the health effect of a radiomics predictor with a condition without a radiomics predictor [15]. However, this RQS point takes second place since has standardization and radiomics models’ validation as a prerequisite.

It should be highlighted that only 20/42 studies refer to IBSI guidelines or used software for radiomic features extraction that are IBSI-compliant (e.g., PyRadiomics). About this topic, it is important to adhere to the standardization of the radiomics features nomenclature and calculation according to the IBSI to improve the reproducibility of scientific research [69]. Future studies are needed in terms of adherence to the standardization of radiomics features.

To our knowledge, this is the first systematic review aimed at exploring whether radiomics could provide information about the DDx of gliomas and evaluating studies by means of an RQS tool.

Previous studies aimed at evaluating the radiomic analysis in different studies for different applications. Park et al. evaluated radiomics analysis in neuro-oncologic studies according to RQS and found that the quality of reporting of radiomics studies was insufficient, with a median RQS of 11 out of 36 [65]. The results of a study by Stanzione et al. on prostate MRI radiomics were in line with our findings and revealed an average RQS score of 7.93 and an RQS percentage of 23% [62]. Wang et al. performed a systematic review of radiomic studies focused on lymphoma and found a mean percentage RQS of 14.2% [70]. Notably, their study included 12 studies also evaluated in our systematic review, in particular those on DDx of glioma and PCNSL. 

Unlike most studies aimed at investigating the quality of radiomic studies by means of RQS, we considered it appropriate to investigate the possible association between RQS and scientometric indexes and found that publications with higher RQS were published in journals with higher IF. However, studies with high/low RQS and low/high IF were also found. Interestingly, we also found included studies’ quality increased with the increasing number of included patients and the number of extracted features.

Our review of the literature has some limitations that should be acknowledged. First, as also highlighted in previous studies, the RQS scoring system is not a gold standard to qualify radiomics studies and still needs revisions to become a widely accepted tool in radiology. Therefore, some aspects of the RQS scoring system such as the difficulty in implementing imaging at multiple time points and phantom study on all scanners, as well as the lack of specificity for a particular study aim, could lower the current literature more than necessary [65,71]. Another limitation affecting our study is that almost all included studies were retrospective, and they are supposed to be more bias-affected [72,73]. This aspect, together with the absence of external validation cohorts for almost all included studies, as well as the comparison with reference standards, prevented us from drawing conclusions about the efficacy of radiomics for glioma DDx. Moreover, the high variability in sample size, inclusion criteria, and methodological settings across studies prevented us from performing a meta-analysis according to the aims of the studies. Moreover, we did not investigate specific radiomics features shared among different studies (according to the specific aim), given the extreme variability of imaging protocol and software for feature extraction.

## 5. Conclusions

Despite promising and encouraging results found in each of the included studies, our study revealed that the current literature on radiomics for glioma DDx still lack the quality required to allow its introduction into clinical practice. In particular, validation is necessary using an external dataset, and improvements need to be made to feature reproducibility, analysis of the clinical utility, pursuits of a higher level of evidence in study design, and openness of science. However, their value might go beyond what was formally assessed with the RQS tool, and further efforts are warranted to provide more solid evidence and the basis for future investigations in this field. This work could provide new insights and help to reach a consensus on the use of the radiomic approach for glioma DDx.

## Supplementary Materials

The following supporting information can be downloaded at: https://www.mdpi.com/article/10.3390/cancers14112731/s1, Table S1: RQS checkpoints, items and points for each item; Table S2: Journal metrics of the included studies; Table S3 Details of methodological quality assessment by Radiomic quality score (RQS) tool.

## References

[1] Mesfin F.B., Al-Dhahir M.A. (2022). Gliomas. StatPearls.

[2] Louis D.N., Perry A., Reifenberger G., von Deimling A., Figarella-Branger D., Cavenee W.K., Ohgaki H., Wiestler O.D., Kleihues P., Ellison D.W. (2016). The 2016 World Health Organization Classification of Tumors of the Central Nervous System: A Summary. Acta Neuropathol..

[3] Sung H., Ferlay J., Siegel R.L., Laversanne M., Soerjomataram I., Jemal A., Bray F. (2021). Global Cancer Statistics 2020: GLOBOCAN Estimates of Incidence and Mortality Worldwide for 36 Cancers in 185 Countries. CA Cancer J. Clin..

[4] Wei R.-L., Wei X.-T. (2021). Advanced Diagnosis of Glioma by Using Emerging Magnetic Resonance Sequences. Front. Oncol..

[5] Gokden M. (2017). If It Is Not a Glioblastoma, Then What Is It? A Differential Diagnostic Review. Adv. Anat. Pathol..

[6] Jacobs A.H., Kracht L.W., Gossmann A., Rüger M.A., Thomas A.V., Thiel A., Herholz K. (2005). Imaging in Neurooncology. Neurotherapeutics.

[7] Carrete L.R., Young J.S., Cha S. (2022). Advanced Imaging Techniques for Newly Diagnosed and Recurrent Gliomas. Front. Neurosci..

[8] Brancato V., Nuzzo S., Tramontano L., Condorelli G., Salvatore M., Cavaliere C. (2020). Predicting Survival in Glioblastoma Patients Using Diffusion MR Imaging Metrics—A Systematic Review. Cancers.

[9] Overcast W.B., Davis K.M., Ho C.Y., Hutchins G.D., Green M.A., Graner B.D., Veronesi M.C. (2021). Advanced Imaging Techniques for Neuro-Oncologic Tumor Diagnosis, with an Emphasis on PET-MRI Imaging of Malignant Brain Tumors. Curr. Oncol. Rep..

[10] Deckert M., Brunn A., Montesinos-Rongen M., Terreni M.R., Ponzoni M. (2014). Primary Lymphoma of the Central Nervous System—A Diagnostic Challenge. Hematol. Oncol..

[11] Fordham A.-J., Hacherl C.-C., Patel N., Jones K., Myers B., Abraham M., Gendreau J. (2021). Differentiating Glioblastomas from Solitary Brain Metastases: An Update on the Current Literature of Advanced Imaging Modalities. Cancers.

[12] Han Y., Yang Y., Shi Z., Zhang A., Yan L., Hu Y., Feng L., Ma J., Wang W., Cui G. (2021). Distinguishing Brain Inflammation from Grade II Glioma in Population without Contrast Enhancement: A Radiomics Analysis Based on Conventional MRI. Eur. J. Radiol..

[13] Jekel L., Brim W.R., von Reppert M., Staib L., Cassinelli Petersen G., Merkaj S., Subramanian H., Zeevi T., Payabvash S., Bousabarah K. (2022). Machine Learning Applications for Differentiation of Glioma from Brain Metastasis—A Systematic Review. Cancers.

[14] Bapuraj J.R., Wang N., Srinivasan A., Rao A. (2021). Advanced Imaging and Computational Techniques for the Diagnostic and Prognostic Assessment of Malignant Gliomas. Cancer J..

[15] Lambin P., Leijenaar R.T.H., Deist T.M., Peerlings J., de Jong E.E.C., van Timmeren J., Sanduleanu S., Larue R.T.H.M., Even A.J.G., Jochems A. (2017). Radiomics: The Bridge between Medical Imaging and Personalized Medicine. Nat. Rev. Clin. Oncol..

[16] Lambin P., Rios-Velazquez E., Leijenaar R., Carvalho S., van Stiphout R.G.P.M., Granton P., Zegers C.M.L., Gillies R., Boellard R., Dekker A. (2012). Radiomics: Extracting More Information from Medical Images Using Advanced Feature Analysis. Eur. J. Cancer.

[17] Rizzo S., Botta F., Raimondi S., Origgi D., Fanciullo C., Morganti A.G., Bellomi M. (2018). Radiomics: The Facts and the Challenges of Image Analysis. Eur. Radiol. Exp..

[18] van Timmeren J.E., Cester D., Tanadini-Lang S., Alkadhi H., Baessler B. (2020). Radiomics in Medical Imaging—“How-to” Guide and Critical Reflection. Insights Imaging.

[19] Lohmann P., Galldiks N., Kocher M., Heinzel A., Filss C.P., Stegmayr C., Mottaghy F.M., Fink G.R., Jon Shah N., Langen K.-J. (2021). Radiomics in Neuro-Oncology: Basics, Workflow, and Applications. Methods.

[20] Ortiz-Ramón R., Ruiz-España S., Mollá-Olmos E., Moratal D. (2020). Glioblastomas and Brain Metastases Differentiation Following an MRI Texture Analysis-Based Radiomics Approach. Phys. Med..

[21] Kim Y., Cho H., Kim S.T., Park H., Nam D., Kong D.-S. (2018). Radiomics Features to Distinguish Glioblastoma from Primary Central Nervous System Lymphoma on Multi-Parametric MRI. Neuroradiology.

[22] Page M.J., McKenzie J.E., Bossuyt P.M., Boutron I., Hoffmann T.C., Mulrow C.D., Shamseer L., Tetzlaff J.M., Akl E.A., Brennan S.E. (2021). The PRISMA 2020 Statement: An Updated Guideline for Reporting Systematic Reviews. BMJ.

[23] Kong Z., Jiang C., Zhu R., Feng S., Wang Y., Li J., Chen W., Liu P., Zhao D., Ma W. (2019). 18F-FDG-PET-Based Radiomics Features to Distinguish Primary Central Nervous System Lymphoma from Glioblastoma. NeuroImage Clin..

[24] Zhang L., Yao R., Gao J., Tan D., Yang X., Wen M., Wang J., Xie X., Liao R., Tang Y. (2021). An Integrated Radiomics Model Incorporating Diffusion-Weighted Imaging and 18F-FDG PET Imaging Improves the Performance of Differentiating Glioblastoma from Solitary Brain Metastases. Front. Oncol..

[25] Mărginean L., Ștefan P.A., Lebovici A., Opincariu I., Csutak C., Lupean R.A., Coroian P.A., Suciu B.A. (2022). CT in the Differentiation of Gliomas from Brain Metastases: The Radiomics Analysis of the Peritumoral Zone. Brain Sci..

[26] Priya S., Liu Y., Ward C., Le N.H., Soni N., Pillenahalli Maheshwarappa R., Monga V., Zhang H., Sonka M., Bathla G. (2021). Radiomic Based Machine Learning Performance for a Three Class Problem in Neuro-Oncology: Time to Test the Waters?. Cancers.

[27] Choi Y.S., Lee H.-J., Ahn S.S., Chang J.H., Kang S.-G., Kim E.H., Kim S.H., Lee S.-K. (2017). Primary Central Nervous System Lymphoma and Atypical Glioblastoma: Differentiation Using the Initial Area under the Curve Derived from Dynamic Contrast-Enhanced MR and the Apparent Diffusion Coefficient. Eur. Radiol..

[28] Alcaide-Leon P., Dufort P., Geraldo A.F., Alshafai L., Maralani P.J., Spears J., Bharatha A. (2017). Differentiation of Enhancing Glioma and Primary Central Nervous System Lymphoma by Texture-Based Machine Learning. AJNR Am. J. Neuroradiol..

[29] Chen Y., Li Z., Wu G., Yu J., Wang Y., Lv X., Ju X., Chen Z. (2018). Primary Central Nervous System Lymphoma and Glioblastoma Differentiation Based on Conventional Magnetic Resonance Imaging by High-Throughput SIFT Features. Int. J. Neurosci..

[30] Wu G., Chen Y., Wang Y., Yu J., Lv X., Ju X., Shi Z., Chen L., Chen Z. (2018). Sparse Representation-Based Radiomics for the Diagnosis of Brain Tumors. IEEE Trans. Med. Imaging.

[31] Artzi M., Bressler I., Ben Bashat D. (2019). Differentiation between Glioblastoma, Brain Metastasis and Subtypes Using Radiomics Analysis: Radiomics Classification of Brain Tumors. J. Magn. Reson. Imaging.

[32] Kang D., Park J.E., Kim Y.-H., Kim J.H., Oh J.Y., Kim J., Kim Y., Kim S.T., Kim H.S. (2018). Diffusion Radiomics as a Diagnostic Model for Atypical Manifestation of Primary Central Nervous System Lymphoma: Development and Multicenter External Validation. Neuro-Oncology.

[33] Kunimatsu A., Kunimatsu N., Kamiya K., Watadani T., Mori H., Abe O. (2018). Comparison between Glioblastoma and Primary Central Nervous System Lymphoma Using MR Image-Based Texture Analysis. MRMS.

[34] Nakagawa M., Nakaura T., Namimoto T., Kitajima M., Uetani H., Tateishi M., Oda S., Utsunomiya D., Makino K., Nakamura H. (2018). Machine Learning Based on Multi-Parametric Magnetic Resonance Imaging to Differentiate Glioblastoma Multiforme from Primary Cerebral Nervous System Lymphoma. Eur. J. Radiol..

[35] Suh H.B., Choi Y.S., Bae S., Ahn S.S., Chang J.H., Kang S.-G., Kim E.H., Kim S.H., Lee S.-K. (2018). Primary Central Nervous System Lymphoma and Atypical Glioblastoma: Differentiation Using Radiomics Approach. Eur. Radiol..

[36] Xiao D.-D., Yan P.-F., Wang Y.-X., Osman M.S., Zhao H.-Y. (2018). Glioblastoma and Primary Central Nervous System Lymphoma: Preoperative Differentiation by Using MRI-Based 3D Texture Analysis. Clin. Neurol. Neurosurg..

[37] Bao S., Watanabe Y., Takahashi H., Tanaka H., Arisawa A., Matsuo C., Wu R., Fujimoto Y., Tomiyama N. (2019). Differentiating between Glioblastoma and Primary CNS Lymphoma Using Combined Whole-Tumor Histogram Analysis of the Normalized Cerebral Blood Volume and the Apparent Diffusion Coefficient. MRMS.

[38] Chen C., Ou X., Wang J., Guo W., Ma X. (2019). Radiomics-Based Machine Learning in Differentiation Between Glioblastoma and Metastatic Brain Tumors. Front. Oncol..

[39] Dong F., Li Q., Jiang B., Zhu X., Zeng Q., Huang P., Chen S., Zhang M. (2020). Differentiation of Supratentorial Single Brain Metastasis and Glioblastoma by Using Peri-Enhancing Oedema Region–Derived Radiomic Features and Multiple Classifiers. Eur. Radiol..

[40] Kunimatsu A., Kunimatsu N., Yasaka K., Akai H., Kamiya K., Watadani T., Mori H., Abe O. (2019). Machine Learning-Based Texture Analysis of Contrast-Enhanced MR Imaging to Differentiate between Glioblastoma and Primary Central Nervous System Lymphoma. MRMS.

[41] Petrujkić K., Milošević N., Rajković N., Stanisavljević D., Gavrilović S., Dželebdžić D., Ilić R., Di Ieva A., Maksimović R. (2019). Computational Quantitative MR Image Features—A Potential Useful Tool in Differentiating Glioblastoma from Solitary Brain Metastasis. Eur. J. Radiol..

[42] Qian Z., Li Y., Wang Y., Li L., Li R., Wang K., Li S., Tang K., Zhang C., Fan X. (2019). Differentiation of Glioblastoma from Solitary Brain Metastases Using Radiomic Machine-Learning Classifiers. Cancer Lett..

[43] Wang B.-T., Liu M.-X., Chen Z.-Y. (2019). Differential Diagnostic Value of Texture Feature Analysis of Magnetic Resonance T2 Weighted Imaging between Glioblastoma and Primary Central Neural System Lymphoma. Chin. Med. Sci. J..

[44] Yun J., Park J.E., Lee H., Ham S., Kim N., Kim H.S. (2019). Radiomic Features and Multilayer Perceptron Network Classifier: A Robust MRI Classification Strategy for Distinguishing Glioblastoma from Primary Central Nervous System Lymphoma. Sci. Rep..

[45] Bae S., An C., Ahn S.S., Kim H., Han K., Kim S.W., Park J.E., Kim H.S., Lee S.-K. (2020). Robust Performance of Deep Learning for Distinguishing Glioblastoma from Single Brain Metastasis Using Radiomic Features: Model Development and Validation. Sci. Rep..

[46] Dastmalchian S., Kilinc O., Onyewadume L., Tippareddy C., McGivney D., Ma D., Griswold M., Sunshine J., Gulani V., Barnholtz-Sloan J.S. (2021). Radiomic Analysis of Magnetic Resonance Fingerprinting in Adult Brain Tumors. Eur. J. Nucl. Med. Mol. Imaging.

[47] Chen C., Zheng A., Ou X., Wang J., Ma X. (2020). Comparison of Radiomics-Based Machine-Learning Classifiers in Diagnosis of Glioblastoma from Primary Central Nervous System Lymphoma. Front. Oncol..

[48] Dong J., Li L., Liang S., Zhao S., Zhang B., Meng Y., Zhang Y., Li S. (2021). Differentiation Between Ependymoma and Medulloblastoma in Children with Radiomics Approach. Acad. Radiol..

[49] Xia W., Hu B., Li H., Geng C., Wu Q., Yang L., Yin B., Gao X., Li Y., Geng D. (2021). Multiparametric-MRI-Based Radiomics Model for Differentiating Primary Central Nervous System Lymphoma from Glioblastoma: Development and Cross-Vendor Validation. J. Magn. Reson. Imaging.

[50] Zhou H., Hu R., Tang O., Hu C., Tang L., Chang K., Shen Q., Wu J., Zou B., Xiao B. (2020). Automatic Machine Learning to Differentiate Pediatric Posterior Fossa Tumors on Routine MR Imaging. AJNR Am. J. Neuroradiol..

[51] Csutak C., Ștefan P.-A., Lenghel L.M., Moroșanu C.O., Lupean R.-A., Șimonca L., Mihu C.M., Lebovici A. (2020). Differentiating High-Grade Gliomas from Brain Metastases at Magnetic Resonance: The Role of Texture Analysis of the Peritumoral Zone. Brain Sci..

[52] Xia W., Hu B., Li H., Shi W., Tang Y., Yu Y., Geng C., Wu Q., Yang L., Yu Z. (2021). Deep Learning for Automatic Differential Diagnosis of Primary Central Nervous System Lymphoma and Glioblastoma: Multi-Parametric Magnetic Resonance Imaging Based Convolutional Neural Network Model. Magn. Reson. Imaging.

[53] Bathla G., Priya S., Liu Y., Ward C., Le N.H., Soni N., Maheshwarappa R.P., Monga V., Zhang H., Sonka M. (2021). Radiomics-Based Differentiation between Glioblastoma and Primary Central Nervous System Lymphoma: A Comparison of Diagnostic Performance across Different MRI Sequences and Machine Learning Techniques. Eur. Radiol..

[54] De Causans A., Carré A., Roux A., Tauziède-Espariat A., Ammari S., Dezamis E., Dhermain F., Reuzé S., Deutsch E., Oppenheim C. (2021). Development of a Machine Learning Classifier Based on Radiomic Features Extracted from Post-Contrast 3D T1-Weighted MR Images to Distinguish Glioblastoma From Solitary Brain Metastasis. Front. Oncol..

[55] Han Y., Zhang L., Niu S., Chen S., Yang B., Chen H., Zheng F., Zang Y., Zhang H., Xin Y. (2021). Differentiation Between Glioblastoma Multiforme and Metastasis from the Lungs and Other Sites Using Combined Clinical/Routine MRI Radiomics. Front. Cell Dev. Biol..

[56] Priya S., Liu Y., Ward C., Le N.H., Soni N., Pillenahalli Maheshwarappa R., Monga V., Zhang H., Sonka M., Bathla G. (2021). Machine Learning Based Differentiation of Glioblastoma from Brain Metastasis Using MRI Derived Radiomics. Sci. Rep..

[57] Priya S., Ward C., Locke T., Soni N., Maheshwarappa R.P., Monga V., Agarwal A., Bathla G. (2021). Glioblastoma and Primary Central Nervous System Lymphoma: Differentiation Using MRI Derived First-Order Texture Analysis—A Machine Learning Study. Neuroradiol. J..

[58] Sartoretti E., Sartoretti T., Wyss M., Reischauer C., van Smoorenburg L., Binkert C.A., Sartoretti-Schefer S., Mannil M. (2021). Amide Proton Transfer Weighted (APTw) Imaging Based Radiomics Allows for the Differentiation of Gliomas from Metastases. Sci. Rep..

[59] Su C.-Q., Chen X.-T., Duan S.-F., Zhang J.-X., You Y.-P., Lu S.-S., Hong X.-N. (2021). A Radiomics-Based Model to Differentiate Glioblastoma from Solitary Brain Metastases. Clin. Radiol..

[60] Xiao D., Wang J., Wang X., Fu P., Zhao H., Yan P., Jiang X. (2021). Distinguishing Brain Abscess from Necrotic Glioblastoma Using MRI-Based Intranodular Radiomic Features and Peritumoral Edema/Tumor Volume Ratio. J. Integr. Neurosci..

[61] Bo L., Zhang Z., Jiang Z., Yang C., Huang P., Chen T., Wang Y., Yu G., Tan X., Cheng Q. (2021). Differentiation of Brain Abscess from Cystic Glioma Using Conventional MRI Based on Deep Transfer Learning Features and Hand-Crafted Radiomics Features. Front. Med..

[62] Stanzione A., Gambardella M., Cuocolo R., Ponsiglione A., Romeo V., Imbriaco M. (2020). Prostate MRI Radiomics: A Systematic Review and Radiomic Quality Score Assessment. Eur. J. Radiol..

[63] Granzier R.W.Y., van Nijnatten T.J.A., Woodruff H.C., Smidt M.L., Lobbes M.B.I. (2019). Exploring Breast Cancer Response Prediction to Neoadjuvant Systemic Therapy Using MRI-Based Radiomics: A Systematic Review. Eur. J. Radiol..

[64] Ursprung S., Beer L., Bruining A., Woitek R., Stewart G.D., Gallagher F.A., Sala E. (2020). Radiomics of Computed Tomography and Magnetic Resonance Imaging in Renal Cell Carcinoma—A Systematic Review and Meta-Analysis. Eur. Radiol..

[65] Park J.E., Kim H.S., Kim D., Park S.Y., Kim J.Y., Cho S.J., Kim J.H. (2020). A Systematic Review Reporting Quality of Radiomics Research in Neuro-Oncology: Toward Clinical Utility and Quality Improvement Using High-Dimensional Imaging Features. BMC Cancer.

[66] Park J.E., Park S.Y., Kim H.J., Kim H.S. (2019). Reproducibility and Generalizability in Radiomics Modeling: Possible Strategies in Radiologic and Statistical Perspectives. Korean J. Radiol..

[67] Vesteghem C., Brøndum R.F., Sønderkær M., Sommer M., Schmitz A., Bødker J.S., Dybkær K., El-Galaly T.C., Bøgsted M. (2020). Implementing the FAIR Data Principles in Precision Oncology: Review of Supporting Initiatives. Brief. Bioinform..

[68] Hasselbring W., Carr L., Hettrick S., Packer H., Tiropanis T. (2020). From FAIR Research Data toward FAIR and Open Research Software. IT Inform. Technol..

[69] Zwanenburg A., Vallières M., Abdalah M.A., Aerts H.J.W.L., Andrearczyk V., Apte A., Ashrafinia S., Bakas S., Beukinga R.J., Boellaard R. (2020). The Image Biomarker Standardization Initiative: Standardized Quantitative Radiomics for High-Throughput Image-Based Phenotyping. Radiology.

[70] Wang H., Zhou Y., Li L., Hou W., Ma X., Tian R. (2020). Current Status and Quality of Radiomics Studies in Lymphoma: A Systematic Review. Eur. Radiol..

[71] Sanduleanu S., Woodruff H.C., de Jong E.E.C., van Timmeren J.E., Jochems A., Dubois L., Lambin P. (2018). Tracking Tumor Biology with Radiomics: A Systematic Review Utilizing a Radiomics Quality Score. Radiother. Oncol..

[72] Norvell D. (2010). Study Types and Bias—Don’t Judge a Study by the Abstract’s Conclusion Alone. Evid. Based Spine-Care J..

[73] Tripepi G., Jager K.J., Dekker F.W., Zoccali C. (2010). Selection Bias and Information Bias in Clinical Research. Nephron Clin. Pract..

