# Supplementary material for: A Systematic Review of the Current Status and Quality of Radiomics for Glioma Differential Diagnosis
Source: Cancers (Basel). 2022 May 31;14(11):2731. doi: 10.3390/cancers14112731 (PMC9179305; doi:10.3390/cancers14112731)
Supplement: Supplementary file 1 [file cancers-14-02731-s001.zip › cancers-1725365-supplementary.pdf]

Supplementary Material

A systematic review on the current status and quality of radiomics for glioma differential diagnosis.

Valentina Brancato[1], Marco Cerrone[1,*], Maria Luisa Lavitrano[2], Marco Salvatore[1], Carlo Cavaliere[1]

[1]IRCCS Synlab SDN, via E.Gianturco 113, 80143, Naples, Italy.

[2] School of Medicine and Surgery, University of Milano-Bicocca, via Cadore 48, 20900 Monza, Italy

S1. Key Search Terms

- (“radiomics” OR “texture” OR “histogram”) AND (“Glioma”) AND (“Diagnosis”)
- (“radiomics” OR “texture” OR “histogram”) AND (“Glioma”) AND (“Differential Diagnosis”)
- (“radiomics” OR “texture” OR “histogram”) AND (“MRI” OR “Magnetic Resonance Imaging”) AND (“Glioma”)
- (“radiomics” OR “texture” OR “histogram”) AND (“CT” OR “Computed Tomography”) AND (“Glioma”)
- (“radiomics” OR “texture” OR “histogram”) AND (“PET” OR “Positron Emission Tomography”) AND (“Glioma”)
- (“radiomics” OR “texture” OR “histogram”) AND (“MRI” OR “Magnetic Resonance Imaging”) AND (“Glioma”) AND (“Diagnosis”)
- (“radiomics” OR “texture” OR “histogram”) AND (“CT” OR “Computed Tomography”) AND (“Glioma”) AND (“Diagnosis”)
- (“radiomics” OR “texture” OR “histogram”) AND (“PET” OR “Positron Emission Tomography”) AND (“Glioma”) AND (“Diagnosis”)
- (“radiomics” OR “texture” OR “histogram”) AND (“Glioma”) AND (“PCNSL” OR “Primary CNS lymphoma” OR “primary central nervous system lymphoma”)
- (“radiomics” OR “texture” OR “histogram”) AND (“Glioma”) AND (“metastasis” OR “metastases”)
- (“radiomics” OR “texture” OR “histogram”) AND (“Glioma”) AND (“brain inflammation”)
- (“radiomics” OR “texture” OR “histogram”) AND (“Glioma”) AND (“brain abscess”)
- (“radiomics” OR “texture” OR “histogram”) AND (“Glioma”) AND (“brain disease”)

S2. PRISMA Checklist

Supplementary Material

Section and Topic	Item #	Checklist item	Location where item is reported
**TITLE**			
Title	1	Identify the report as a systematic review.	1
**ABSTRACT**			
Abstract	2	See the PRISMA 2020 for Abstracts checklist.	1
**INTRODUCTION**			
Rationale	3	Describe the rationale for the review in the context of existing knowledge.	2
Objectives	4	Provide an explicit statement of the objective(s) or question(s) the review addresses.	3
**METHODS**			
Eligibility criteria	5	Specify the inclusion and exclusion criteria for the review and how studies were grouped for the syntheses.	3
Information sources	6	Specify all databases, registers, websites, organisations, reference lists and other sources searched or consulted to identify studies. Specify the date when each source was last searched or consulted.	3
Search strategy	7	Present the full search strategies for all databases, registers and websites, including any filters and limits used.	3
Selection process	8	Specify the methods used to decide whether a study met the inclusion criteria of the review, including how many reviewers screened each record and each report retrieved, whether they worked independently, and if applicable, details of automation tools used in the process.	3-4
Data collection process	9	Specify the methods used to collect data from reports, including how many reviewers collected data from each report, whether they worked independently, any processes for obtaining or confirming data from study investigators, and if applicable, details of automation tools used in the process.	3-4
Data items	10a	List and define all outcomes for which data were sought. Specify whether all results that were compatible with each outcome domain in each study were sought (e.g. for all measures, time points, analyses), and if not, the methods used to decide which results to collect.	4
	10b	List and define all other variables for which data were sought (e.g. participant and intervention characteristics, funding sources). Describe any assumptions made about any missing or unclear information.	4
Study risk of bias assessment	11	Specify the methods used to assess risk of bias in the included studies, including details of the tool(s) used, how many reviewers assessed each study and whether they worked independently, and if applicable, details of automation tools used in the process.	4
Effect measures	12	Specify for each outcome the effect measure(s) (e.g. risk ratio, mean difference) used in the synthesis or presentation of results.	NA
Synthesis methods	13a	Describe the processes used to decide which studies were eligible for each synthesis (e.g. tabulating the study intervention characteristics and comparing against the planned groups for each synthesis (item #5)).	NA
	13b	Describe any methods required to prepare the data for presentation or synthesis, such as handling of missing summary	NA

Section and Topic	Item #	Checklist item	Location where item is reported
		statistics, or data conversions.	
	13c	Describe any methods used to tabulate or visually display results of individual studies and syntheses.	NA
	13d	Describe any methods used to synthesize results and provide a rationale for the choice(s). If meta-analysis was performed, describe the model(s), method(s) to identify the presence and extent of statistical heterogeneity, and software package(s) used.	NA
	13e	Describe any methods used to explore possible causes of heterogeneity among study results (e.g. subgroup analysis, meta-regression).	NA
	13f	Describe any sensitivity analyses conducted to assess robustness of the synthesized results.	NA
Reporting bias assessment	14	Describe any methods used to assess risk of bias due to missing results in a synthesis (arising from reporting biases).	NA
Certainty assessment	15	Describe any methods used to assess certainty (or confidence) in the body of evidence for an outcome.	NA
**RESULTS**			
Study selection	16a	Describe the results of the search and selection process, from the number of records identified in the search to the number of studies included in the review, ideally using a flow diagram.	4-5
	16b	Cite studies that might appear to meet the inclusion criteria, but which were excluded, and explain why they were excluded.	4-5
Study characteristics	17	Cite each included study and present its characteristics.	5-14, Table 1
Risk of bias in studies	18	Present assessments of risk of bias for each included study.	NA
Results of individual studies	19	For all outcomes, present, for each study: (a) summary statistics for each group (where appropriate) and (b) an effect estimate and its precision (e.g. confidence/credible interval), ideally using structured tables or plots.	NA
Results of syntheses	20a	For each synthesis, briefly summarise the characteristics and risk of bias among contributing studies.	14-15
	20b	Present results of all statistical syntheses conducted. If meta-analysis was done, present for each the summary estimate and its precision (e.g. confidence/credible interval) and measures of statistical heterogeneity. If comparing groups, describe the direction of the effect.	15
	20c	Present results of all investigations of possible causes of heterogeneity among study results.	NA
	20d	Present results of all sensitivity analyses conducted to assess the robustness of the synthesized results.	NA
Reporting biases	21	Present assessments of risk of bias due to missing results (arising from reporting biases) for each synthesis assessed.	NA
Certainty of evidence	22	Present assessments of certainty (or confidence) in the body of evidence for each outcome assessed.	NA
**DISCUSSION**			

Section and Topic	Item #	Checklist item	Location where item is reported
Discussion	23a	Provide a general interpretation of the results in the context of other evidence.	15-16
	23b	Discuss any limitations of the evidence included in the review.	16
	23c	Discuss any limitations of the review processes used.	16
	23d	Discuss implications of the results for practice, policy, and future research.	16
**OTHER INFORMATION**			
Registration and protocol	24a	Provide registration information for the review, including register name and registration number, or state that the review was not registered.	NA
	24b	Indicate where the review protocol can be accessed, or state that a protocol was not prepared.	NA
	24c	Describe and explain any amendments to information provided at registration or in the protocol.	NA
Support	25	Describe sources of financial or non-financial support for the review, and the role of the funders or sponsors in the review.	NA
Competing interests	26	Declare any competing interests of review authors.	18
Availability of data, code and other materials	27	Report which of the following are publicly available and where they can be found: template data collection forms; data extracted from included studies; data used for all analyses; analytic code; any other materials used in the review.	NA

From: Page MJ, McKenzie JE, Bossuyt PM, Boutron I, Hoffmann TC, Mulrow CD, et al. The PRISMA 2020 statement: an updated guideline for reporting systematic reviews. BMJ 2021;372:n71. doi: 10.1136/bmj.n71

S3. Radiomics Quality Score items

Table S1. RQS checkpoints, items and points for each item.

RQS Checkpoint	RQS Item	RQS points
Checkpoint 1	**Image protocol quality** - well-documented image protocols (for example, contrast, slice thickness, energy, etc.) and/or usage of public image protocols allow reproducibility/replicability.	+1 (if protocols are well-documented)<br><br>+1 (if public protocol is used)
Checkpoint 2	**Multiple segmentations** - possible actions are: segmentation by different physicians/algorithms/software, perturbing segmentations by (random) noise, segmentation at different	+1

	breathing cycles. Analyse feature robustness to segmentation variabilities.	
	**Phantom study on all scanners** - detect inter-scanner differences and vendor-dependent features. Analyse feature robustness to these sources of variability.	+1
	**Imaging at multiple time points** - collect images of individuals at additional time points. Analyse feature robustness to temporal variabilities (for example, organ movement, organ expansion/ shrinkage).	+1
Checkpoint 3	**Feature reduction** or adjustment for multiple testing - decreases the risk of overfitting. Overfitting is inevitable if the number of features exceeds the number of samples. Consider feature robustness when selecting features.	-3 (if neither measure is implemented)<br><br>+3 (if either measure is implemented)
	**Multivariable analysis with non radiomics features** (for example, EGFR mutation) - is expected to provide a more holistic model. Permits correlating/inferencing between radiomics and non radiomics features.	+1
	**Detect and discuss biological correlates** - demonstration of phenotypic differences (possibly associated with underlying gene–protein expression patterns) deepens understanding of radiomics and biology.	+1
	**Cut-off analyses** - determine risk groups by either the median, a previously published cut-off or report a continuous risk variable. Reduces the risk of reporting overly optimistic results.	+1

	**Discrimination statistics** - report discrimination statistics (for example, C-statistic, ROC curve, AUC) and their statistical significance (for example, p-values, confidence intervals). One can also apply resampling method (for example, bootstrapping, cross-validation).	+1 (if a discrimination statistic and its statistical significance are reported)<br><br>+1 (if a resampling method technique is also applied)
	**Calibration statistics** - report calibration statistics (for example, Calibration-in-the-large/slope, calibration plots) and their statistical significance (for example, P-values, confidence intervals). One can also apply resampling method (for example, bootstrapping, cross-validation).	+1 (if a calibration statistic and its statistical significance are reported)<br><br>+1 (if a resampling method technique is also applied)
	**Prospective study registered in a trial database** - provides the highest level of evidence supporting the clinical validity and usefulness of the radiomics biomarker.	+7 (for prospective validation of a radiomics signature in an appropriate trial)
	**Validation** - the validation is performed without retraining and without adaptation of the cut-off value, provides crucial information with regard to credible clinical performance.	-5 (if validation is missing)<br><br>+2 (if validation is based on a dataset from the same institute)<br><br>+3 (if validation is based on a dataset from another institute)<br><br>+4 (if validation is based on two datasets from two distinct institutes)<br><br>+4 (if the study validates a previously published signature)

		+5 (if validation is based on three or more datasets from distinct institutes)<br><br>*Datasets should be of comparable size and should have at least 10 events per model feature.
	**Comparison to 'gold standard'** - assess the extent to which the model agrees with/is superior to the current 'gold standard' method (for example, TNM-staging for survival prediction). This comparison shows the added value of radiomics.	+2
	**Potential clinical utility** - report on the current and potential application of the model in a clinical setting (for example, decision curve analysis).	+2
	**Cost-effectiveness analysis** - report on the cost-effectiveness of the clinical application (for example, QALYs generated).	+1
	**Open science and data** - make code and data publicly available. Open science facilitates knowledge transfer and reproducibility of the study.	+1 (if scans are open source)<br><br>+1 (if region of interest segmentations are open source)<br><br>+1 (if code is open source)<br><br>+1 (if radiomics features are calculated on a set of representative ROIs and the calculated features and representative ROIs are open source

Supplementary Material

S4. Journal Metrics

Table S2. Journal metrics of the included studies.

Authors, Year	Journal	IF of the year of publication	5 Year IF	CiteScore	H-index	H-index first author	H-index first author no self citations
Choi et al., 2016	European Radiology	3.967	4.714	4.08	134	9	9
Alcaide-Leon et al., 2017	AJNR	3.653	3.268	3.28	162	8	8
Chen et al., 2017	International Journal of Neuroscience	1.848	1.96	1.58	57	14	14
Wu et al., 2017	IEEE - Transactions on Medical Imaging	6.131	13.308	8.58	195	1	1
Artzi et al., 2019	JOURNAL OF MAGNETIC RESONANCE IMAGING	3.732	4.475	7.8	160	17	17
Kang et al., 2018	Neuro-oncology	9.384	7.7	16.1	113	1	1
Kim et al., 2018	Neuroradiology	2.504	2.274	4.0	91	11	11
Kunimatsu., 2018	MRMS	1.455	1.78	1.48	35	28	27
Nakagawa et al., 2018	European Journal of Radiology	2.948	3.279	4.8	109	4	4
Suh et al., 2018	European Radiology	3.962	4.714	6.9	143	3	3
Xiao et al., 2018	Clinical Neurology and Neurosurgery	1.672	1.838	2.7	69	2	2
Bao et al., 2019	MRMS	1.481	1.78	3.2	37	1	1
Chen et al., 2019	FRONTIERS IN ONCOLOGY	4.848	5.729	3.9	83	7	7
Dong et al., 2019	European Radology	5.315	4.714	7.7	149	11	11

Kong et al., 2019	Neuroimage: Clinical	4.35	4.924	7.0	68	9	9
Kumimatsu et al., 2019	MRMS	1.481	1.78	3.2	37	31	31
Petrujkic et al., 2019	European Journal of Radiology	2.687	3.279	4.6	115	1	1
Quian et al., 2019	Cancer letters	6.509	8.033	14.0	182	16	15
Wang et al., 2019	Chinese Medical Sciences Journal	0.533	0.746	1.5	21	4	3
Yun et al., 2019	Scientific Reports	4.011	4.409	7.1	213	4	4
Bae et al., 2020	Scientific Reports	4.379	4.409	7.1	213	9	9
Dastmalchian et al., 2020	Eur J Nucl Med Mol Imaging	9.236	5.618	11.6	163	6	6
Chen et al., 2020	Frontiers in oncology	6.244	5.729	4.6	83	8	8
Dong et al., 2020	Academic Radiology	3.173	2.582	4.7	96	2	2
Oritz-Ramon et al., 2020	Physica Medica	2.685	2.634	4.2	44	4	4
Xia et al., 2020	JMRI	4.813	4.475	7.8	160	11	10
Zhou et al., 2020	AJNR	3.825	3.268	5.8	177	8	8
Csutak et al., 2020	Brain Sciences	3.394	3.703	3.0	44	5	4
Xia et al., 2021	JOURNAL OF MAGNETIC RESONANCE IMAGING	4.813	4.475	7.8	160	11	10
Bathla et al., 2021	European Radiology	5.315	4.714	7.7	149	15	15
Priya et al., 2021	Cancers	6.102	6.275	4.4	76	4	4
De Causans at al.., 2021	FRONTIERS IN ONCOLOGY	6.244	5.729	3.9	83	1	1

Zhang et al.., 2021	FRONTIERS IN ONCOLOGY	6.244	5.729	3.9	83	0	0
Han et al., 2021	European Journal of Radiology	3.528	3.279	4.6	115	11	10
Han et al.., 2021	Frontiers in Cell and Developmental Biology	6.684	6.079	2.7	53	7	7
Priya et al. a, 2021	Scientific Reports	4.379	4.409	7.1	213	4	4
Priya et al. b, 2021	The Neuroradiology Journal	1.512	1.63	2.5	22	4	4
Santoretti et al., 2021	Scientific Reports	4.379	4.409	7.1	213	8	6
Su et al., 2021	Clinical Radiology	2.35	2.247	3.9	90	6	6
Xiao et al., 2021	Journal of integrative Neuroscience	2.117	1.463	1.8	33	4	4
Bo et al., 2021	Frontiers in medicine	11.48	4.557	4.1	39	0	0
Marginean et al., 2022	Brain Sciences	3.114	3.32	3.0	33	1	1

S5. Abbreviations for Table 1

R= retrospective; P= prospective; PCNSL = Primary Central Nervous System Lymphoma; GBM= Glioblastoma Multiforme; MET= metastasis; EP= Ependymoma; MB=meduloblastoma; PA = pilocytic astrocytoma; HGGs= High grade gliomas; NP= Number of Patients; CE-T1WI = Contrast Enanchement T1 Weighted Imaging; AUC= Area under the curve; IAUC= Initial Area Under the Curve; ADC= Apparent diffusion coefficient; T2-WI= T2 weighted imaging; Flair= Fluid Attenuated Inversion Recovery; DWI= diffusion weighted imaging; rCBV= relative cerebral blood volume; SUV = standardized uptake value; NCC= normal controlateral cortex; NBM= normal brain mean; FDG= fluorodeoxyglucose; APTw= Amide Proton Transfer weighted; CT= Computized Tomography; S= semiautomatic; 3D= three dimensions; M= manual; A= automatic; 2D= two dimensions; SEG= segmentation; MIPAV= Medical Imaging Processing Analysis & Visualization; NR= not reported; SIFT= Scale Invariant Feature Transform; LoG= Logdomain wavelet filters; NN= L= Deep Transfer Learning; FS= Feature Selection;

SVM= Support Vector Machine; NCA= Neighborhood component analysis; PCA= Principal Component Analysis; mRMR= minimum redundancy maximum relevance; ICC= Intraclass Correlation Coefficient; RF= Random Forest; GBDT= Gradient Boosting Decision Tree; CFS = Correlation-based Feature Selection; MLP= Multilayer Perceptron; MWW= Mann-Whitney-Wilcoxon; MIC= Maximal Information Coefficient; TPOT= Tree-based pipeline optimization tool; CM= Classification Model ; KNN= k-nearest neighbor; DT= Decision Tree; BoF= Bag of Features; NB= Naïve Bayes; LDA= Linear Discriminant analysis; RF= Random Forest; AB= Adaboost ; LR= Linear Regression ; NN= Neural Network; L-SVM= Linear Kernel; DNN= Deep Neural Network; VM= Validation Method ; LOOCV= Leave One Out Cross Validation; CV= Cross Validation; SRR= Spars representation-based radiomics; CNN= Convolutional Neural Network; BM= Brain Metastasis; IMD= Intracranial Metastatic Disease; HCR= Hand Crafted Radiomics; DTL= Deep Transfer Learning.

S6. Radiomics Quality Score assessment

Table S3. Details of methodological quality assessment by Radiomic quality score (RQS) tool.

Author	Image protocol quality	Multiple segmentations	Phantom study on all scanners	Imaging at multiple time points	Feature reduction or adjustment for multiple testing	Multivariable analysis with non radiomics features	Detect and discuss biological correlates	Cut-off analyses	Discrimination statistics	Calibration statistics	Prospective study registered in a trial database	Validation	Comparison to gold standard	Potential clinical utility	Cost-effectiveness analysis	Open science and data	Total
Choi et al., 2016	1	0	0	0	-3	0	0	1	2	0	0	-5	0	0	0	0	0 (0%)
Alcaide-Leon et al., 2017	1	1	0	0	3	0	0	1	2	0	0	-5	0	0	0	0	3 (8,33%)
Chen et al., 2017	1	1	0	0	3	0	0	1	2	0	7	2	0	2	0	0	19 (52,78%)
Wu et al., 2017	1	0	0	1	3	1	1	0	2	0	0	2	0	0	0	0	11 (30,56%)
Artzi et al., 2019	1	0	0	0	3	0	0	0	2	0	0	2	0	2	0	0	10 (27,78%)
Kang et al., 2018	1	1	0	0	3	1	0	1	2	0	0	3	2	2	0	1	17 (47,22%)
Kim et al., 2018	1	1	0	1	3	0	0	1	2	0	0	3	0	2	0	1	15 (41,67%)
Kunimatsu., 2018	1	1	0	0	3	0	0	0	0	0	0	-5	0	0	0	0	0 (0%)
Nakagawa et al., 2018	1	0	0	1	3	0	0	1	2	0	0	-5	0	2	0	0	5 (13,89%)
Suh et al., 2018	1	1	0	1	3	0	0	1	2	0	0	2	2	2	0	1	16 (44,44%)
Xiao et al., 2018	1	0	0	1	3	0	0	1	2	0	0	-5	0	2	0	0	5 (13,89%)

Bao et al., 2019	1	0	0	0	-3	0	0	0	1	0	0	-5	0	0	0	0	0 (0%)
Chen et al., 2019	2	1	1	0	3	0	0	0	2	0	0	2	0	2	0	1	14 (38,89%)
Dong et al., 2019	1	0	0	0	3	0	0	0	2	0	0	2	0	2	0	0	10 (27,78%)
Kong et al., 2019	1	0	0	0	3	0	0	0	2	0	0	2	2	0	0	0	10 (27,78%)
Kumimatsu et al., 2019	1	0	0	0	3	0	0	1	2	0	0	2	0	2	0	1	12 (33,33%)
Petrujkic et al., 2019	1	0	0	0	-3	0	0	0	2	0	0	-5	0	0	0	0	0 (0%)
Quian et al., 2019	1	1	0	0	3	0	0	0	2	0	0	2	0	0	0	1	10 (27,78%)
Wang et al., 2019	1	1	0	0	-3	0	0	1	1	0	0	-5	0	0	0	0	0 (0%)
Yun et al., 2019	1	0	1	0	3	0	0	1	2	0	0	4	0	2	0	1	15 (41,67%)
Bae et al., 2020	1	1	0	0	3	0	0	0	2	0	0	3	0	2	0	0	12 (33,33%)
Dastmalchian et al., 2020	1	1	0	0	3	0	0	0	2	0	7	-5	0	2	0	0	11 (30,56%)
Chen et al., 2020	1	1	0	0	3	0	1	0	2	0	0	2	0	0	0	1	11 (30,56%)
Dong et al., 2020	1	1	0	0	3	0	0	0	2	0	0	-5	0	2	0	0	4 (11,11%)
Oritz-Ramon et al., 2020	1	0	0	0	3	0	0	0	2	0	0	2	0	2	0	0	10 (27,78%)
Xia et al., 2020	1	1	1	0	3	0	0	1	2	0	0	3	2	2	0	0	16 (44,44%)
Zhou et al., 2020	1	1	0	0	3	0	0	0	2	0	0	2	0	2	0	0	11 (30,56%)
Csutak et al., 2020	1	1	0	0	3	0	0	1	1	0	0	-5	0	0	0	0	2 (5,55%)
Xia et al., 2021	1	1	0	0	3	0	0	0	2	0	0	2	2	2	0	0	13 (36,11%)
Bathla et al., 2021	1	1	0	0	3	0	0	0	2	1	0	-5	0	0	0	0	3 (8,33%)
Priya et al., 2021	1	0	0	0	3	0	0	0	2	0	0	-5	0	0	0	0	1 (2,78%)
De Causans at al.., 2021	1	1	0	0	3	0	0	0	2	0	0	2	0	0	0	0	9 (25%)
Zhang et al.., 2021	1	1	0	0	3	0	0	0	2	0	0	2	0	0	0	0	9 (25%)
Han et al., 2021	1	1	0	0	3	1	0	0	2	1	0	3	2	2	0	0	16 (44,44%)

Han et al., 2021	1	1	0	0	3	0	0	0	2	0	0	2	2	2	0	0	13 (36,11%)
Priya et al. a, 2021	2	0	0	0	3	0	0	0	2	0	0	-5	0	2	0	1	5 (13,89%)
Priya et al. b, 2021	1	0	0	0	3	0	0	0	2	0	0	-5	0	0	0	0	1 (2,78%)
Santoretti et al., 2021	1	0	0	0	3	0	0	0	2	0	0	2	0	0	0	1	9 (25%)
Su et al., 2021	1	1	0	0	3	0	0	0	2	0	0	2	0	0	0	0	9 (25%)
Xiao et al., 2021	1	1	0	0	3	1	0	1	2	0	0	2	0	2	0	0	13 (36,11%)
Bo et al., 2021	1	1	0	0	3	0	0	1	2	0	0	2	2	0	0	3	15 (41,67%)
Marginean et al., 2022	1	0	0	0	3	0	0	1	1	0	0	-5	0	0	0	0	1 (2,78%)
